# ChoRe: A device for trans-catheter chordae tendineae repair

**DOI:** 10.1177/0954411919848856

**Published:** 2019-05-08

**Authors:** Costanza Culmone, Awaz Ali, Marta Scali, Arianna Menciassi, Paul Breedveld

**Affiliations:** 1 Bio-Inspired Technology Group (BITE), Department of BioMechanical Engineering, Faculty of Mechanical, Maritime and Materials Engineering, Delft University of Technology, Delft, The Netherlands; 2 The BioRobotics Institute, Scuola Superiore Sant’Anna, Pisa, Italy

**Keywords:** Mitral valve, repair, regurgitation, chordae tendineae, trans-catheter, additive manufacturing

## Abstract

This work focuses on the design of a new device (called ChoRe) to place artificial chords in the mitral valve structure during a trans-catheter procedure. The aim of the device is to restore the correct functionality of the valve and solve mitral valve regurgitation, that is, a common consequence of chordae tendineae rupture. An analysis of the requirements was carried out and used to design and develop a first functional prototype. The resulting device was able to connect artificial chords at the posterior leaflet of the mitral valve and at the apex of the left ventricle, also allowing the control of the artificial chord length. The ChoRe was tested ex-vivo in bovine hearts. The qualitative assessment of the ChoRe focused on the performance of the device and preliminary evaluation of the procedure time. Results demonstrated that the device is able to create a top and bottom fixation in an average time of 3.45 ± 1.44 min. Future improvements will focus on enhancing the connection at the leaflet, as well as the overall functionality, in order to guarantee better control of the artificial chord length. This work shows future potentials for more patient-specific treatments in trans-catheter scenarios for mitral valve repair.

## Introduction

### Mitral valve regurgitation

**Figure 1.**
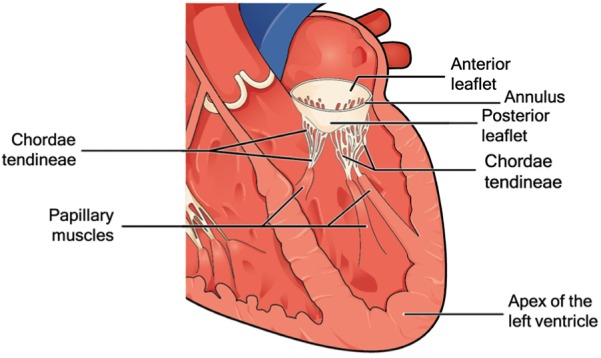
Mitral valve structure with the principal elements: the anterior and posterior leaflet, the annulus, the chordae tendineae, the papillary muscles, and the apex of the left ventricle. *Source*: Adapted from Betts et al.^3^

Heart disease is a leading cause of death in industrialized countries. One of the most prevalent heart valve dysfunctions, which cause disturbed blood flow through the heart, is mitral regurgitation in which blood leaks backward through the mitral valve between the left atrium and the left ventricle.^1^ Mitral regurgitation increases with age and occurs in 10% of people older than 75 years.^2^ Figure 1 shows a cross section of the mitral valve structure with the so-called chordae tendineae, that is, branched chords that connect the mitral valve leaflets to the papillary muscles that form part of the ventricle wall.^4^ One of the main causes of mitral regurgitation is a lengthening of the chordae tendineae, resulting in the valve to open in the wrong direction. In mitral regurgitation, the leaflets of the valve do not close completely and are not able to reach the so-called coaptation, which corresponds to the ideal closure of the valve. Chordae tendineae elongation or breakage occurs for 70% in the posterior leaflet.^5^

### State-of-the-art interventional techniques

Various surgical approaches have been developed to treat mitral regurgitation, ranging from replacing the full valve system to restoring the function by repairing a single element of the valve. During replacement, the diseased mitral valve is substituted by an artificial valve consisting of the annulus and the leaflets made of biological or artificial materials.^6,7^ During repair, single elements of the mitral valve, such as the leaflets or the chordae tendineae, are restored in their original functionalities.^8^ Both the replacement and the repair approach can be conducted via traditional open-heart surgery, with a sternal incision of approximately 240 mm,^9^ or by minimally invasive cardiac surgery (MICS), in which a smaller incision of approximately 75 mm is made under the right breast.^10^

In open-heart surgery, the surgeon can directly reach the target structures, and has freedom of hand movement and a direct view of the operation site. These elements are limited in MICS. In this case, the loss of space and degrees of freedom make the procedure more complex and, in some cases, lead to a longer operative time,^11^ even if many methodologies such as trans-esophageal echocardiography (TEE) guidance ^12,13^ have been developed to help the surgeon in visualizing the operative site. Both open surgery and MICS are performed with the use of cardiopulmonary bypass, a technique that temporarily substitutes the functions of heart and lungs during surgery.

However, due to the invasiveness of the surgery, in almost 50% of patient with mitral valve regurgitation, the surgery is not performed due to the high risk of mortality related to the advanced age and comorbidities.^14^ In this scenario, innovative approaches such as trans-apical (a 45-mm incision to reach the apex)^15^ and trans-catheter (catheters are guided through the blood vessels to reach the target point, and thereby limiting the invasiveness of the procedure for the patient with an incision in the skin only 5–6 mm long)^16,17^ techniques are gaining momentum. Especially in the trans-catheter techniques, significative successful results have been reported in high-risk patients.^18^ Due to a lower level of invasiveness, the patient is generally under local anesthesia in beating-heart condition, thus avoiding long recovery time, post-procedural complications, and lowering risks of infection.^9,12,15^

Even though mitral valve replacement can be achieved successfully, a majority of cardiac surgeons prefer a repair approach in which the valve function is restored with longer durability and without a need for long drug therapy after surgery.^19,20^ Moreover, the damage often involves only one element of the mitral valve, such as the chordae tendineae, which is the one that needs to be repaired. Devices developed and tested, such as the Neochord (Neochord, Inc., St. Luis Park, MN, USA),^21^ TSD-5 (Harpoon Medical, Inc. Baltimore, MD, USA),^22^ V-chordal off-Pump,^23^ and Babic device,^24^ use the trans-apical approach to repair the chordae tendineae. In the trans-apical technique performed by the Neochord, the device is inserted through the apex of the heart to enter the ventricle. Then, the device attaches the artificial chords to the leaflet. Finally, the artificial chords are fixed to the outer wall of the ventricle apex. The trans-apical technique performed by the Neochord device is the only one clinically accepted and is currently in the randomized clinical trial phase.^25^

However, the trans-apical approach still needs an incision in the skin, of approximately 45 mm, and in the heart, to insert the 8-mm instrument.^26^ Moreover, despite promising results in trans-catheter mitral valve implantation (TMVI)^27^ and annulus and leaflet repair,^28–31^ there is no clinically accepted device that is capable of performing the reconstruction of the chordae tendineae via the trans-catheter route.

Therefore, in this work, we design a new device to repair the chordae tendineae, mainly focusing on the working principle of the device to place artificial chords at the required sites in a trans-catheter scenery. This work presents the most distal segment of the entire device. The design of the catheter and the guiding sheath for insertion of the device, as well as the method of positioning the device into the ventricle, are out of our scope at this stage.

## Design of the ChoRe

### Conventional procedure

The technique for the repair of chordae tendineae has continuously changed over the years, but, regardless of the level of invasiveness, the main steps of the procedure are similar in all methods.^32^ The damaged chordae tendineae are left in place and do not need to be removed, while new artificial chords (usually made of artificial biocompatible material) are installed to repair the valve. In the first step, the artificial chords are fixed to the bottom of the ventricle, the papillary muscle, or the apex of the ventricle (see Figure 1). The artificial chords are then connected to the leaflet, their length is adjusted and fixed, and a leakage test is carried out.^33^ The number of artificial chords generally placed comes to a maximum of 10,^34^ with an average of three, depending on the valve defect.^35^ One of the most important factors affecting the end result of the intervention is that the artificial chord length has to be estimated by means of echocardiographic images and adjusted during the procedure depending on the patient’s anatomy. The length of the artificial chords is generally in the range of 14–21 mm^34^ if the chord is attached to the papillary muscles, or 53–85 mm^36^ if the chord is attached to the apex of the ventricle. Moreover, the artificial chords have to be attached to a safe and solid connection to the free edge of the leaflet and the bottom of the ventricle.^37,38^ It is extremely important not to damage the healthy chordae tendineae or the mitral valve, considering that the prolapsing leaflet tissue is very thin and fragile, while the papillary muscle is thick and rather stable.^32^

### Design requirements

The knowledge of the conventional procedure led to a new design for an innovative device capable of repairing the chordae tendineae in a trans-catheter scenario. The general idea behind the device is that it has to be used in combination with a dedicated steerable catheter that enables the device to reach the target site. The device has been designed considering the possibility of performing measurements in a pre-operative phase by means of echocardiographic images, in order to estimate the required length of the artificial chords. We decided to use an anchorage at the apex of the heart due to the difficulties involved in grasping the papillary muscles having a width of approximately 15 mm.^39^ At the start of the design process, we established a set of design requirements:

#### Functions

- The device must connect an artificial chord to the bottom of the ventricle.
- The device must connect an artificial chord to the mitral valve leaflet.
- The device must adjust the length of the artificial chord depending on the patient’s anatomy.

#### Size constraints

- For insertion in the femoral vein, the inferior vena cava, and the heart, the maximum diameter of the device should be 22 F (7.3 mm), as in currently available devices for interventions inside of the heart.^40^
- The rigid length of the device must not exceed 25 mm, considering the average physiological size of an adult human heart and the curvature of the inferior vena cava blood vessel.
- The shape of the device must be smooth without any sharp edges.

**Figure 2.**
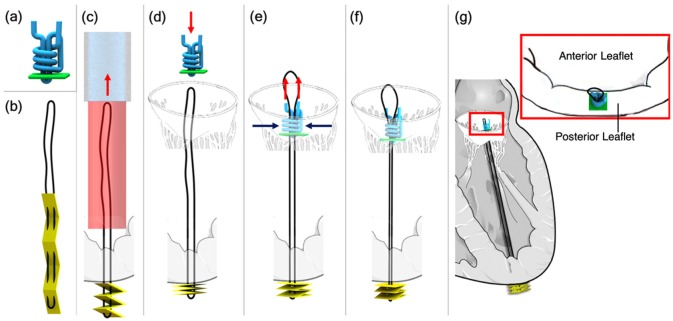
Sketches of the ChoRe implant: (a) The pre-constructed knot with the polyester thread in blue and the leaflet pledget in green; (b) The artificial chord with the ePTFE thread in black and the apex pledget in yellow; (c) The apex pledget, previously positioned into the device, is pushed through the ventricle wall using a cannula to create the apex fixation. The apex pledget folds into an accordion shape due to the movement of the device from the apex to the leaflet and the interaction of the apex pledget with the ventricle wall; (d) the pre-constructed knot is pushed by the surgeon against the leaflet; (e–f) once the length is decided, the surgeon tightens the pre-constructed knot around the artificial chord to finalize the procedure; (g) sketch of the artificial chord installed in the left ventricle, the red box shows a close-up of the implant on the posterior leaflet. The red arrows represent the movements made by the surgeon while the blue arrows the tightening of the pre-constructed knot. The catheter (light blue) and the device (red) are shown only once for simplicity.

The assumption here is that the procedure is performed using TEE and fluoroscopy guidance to visualize the surgical site^41^ and starts right after the insertion of the device mounted on a catheter with a steerable guiding sheath, which has been positioned in the left atrium through a trans-septal puncture. The steerable guiding sheath requires a deflection of approximately 90° to reach the perpendicular position in the left atrium.^42^ Once the device reaches its position perpendicular to the plane of the mitral valve, the device is moved forward in order to reach the apex of the left heart through the left ventricle (Figure 2).

### Overall design

The overall design of our device, called “ChoRe,” was created in Solidworks 2015–2016. Its function relies on first creating the bottom connection at the ventricle wall and then ending with the top connection at the valve leaflet. The procedure can be divided into three main phases: (1) apex fixation, (2) leaflet fixation, and (3) length adjustment. The most important component of the ChoRe is the “implant”: the component that has to fix the regurgitation. The implant is composed out of two elements: an *artificial chord* and a *pre-constructed knot* (Figure 2).

The *artificial chord* is composed of an expanded polytetrafluoroethylene (ePTFE) thread in a loop configuration, black in Figure 2(b), and an apex pledget (a piece of wad made of felt textile material), yellow in Figure 2(b). The apex pledget is sewn with the ePTFE thread in a loop configuration and folded into an accordion shape to allow deployment for creating a stable placement of the apex connection, as presented for a different purpose in Siminiak et al.^29^(Figure 2(c)).

**Figure 3.**
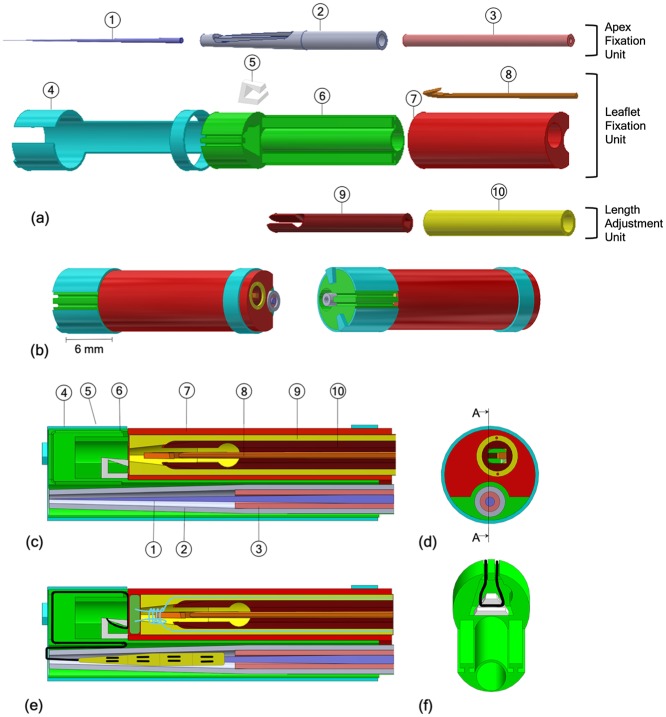
(a) Exploded view of the CAD ChoRe model: (1) Needle, (2) cannula, (3) piston, (4) external shell, (5) thread support, (6–7) leaflet clamp, (8) harpoon-shaped needle, (9) clamping tube, (10) chord grasper; (b) CAD model of the assembled ChoRe device; (c) cross-section view of the CAD ChoRe model; (d) top view of the CAD ChoRe model; (e) cross-section view of the CAD model with the artificial chord, and the pre-constructed knot; (f) a detail of the internal structure of the leaflet clamp and the thread support holding the artificial chord.

The *pre-constructed knot* is composed of a leaflet pledget, green in Figure 2(a), and a polyester thread, blue in Figure 2(a). Inspired by the knot used in Ramponi et al.’s^43^ work, the pre-constructed knot is an adjustable fixation element at the leaflet side. The polyester thread creates a multiple loops knot using the leaflet pledget as support. The knot is tightened around the artificial chord only when the required chord length has been determined by the interventionist (Figure 2(d)–(g)). In addition to the implant, the ChoRe has been designed with 10 components that can be grouped into three units: an apex fixation unit, a leaflet fixation unit, and a length adjustment unit, Figure 3.

#### Apex fixation unit

The apex fixation unit consists of a needle (1), a cannula (2), and a piston (3), Figure 3(a). The piston and the cannula are both hollow and fit around the needle. The cannula, 2.3 mm in diameter, ends in a conical shape with an opening. Before the procedure, the cannula is preloaded with the needle, surrounded by the artificial chord. In the first step of the procedure, the needle is inserted through the apex of the heart to define the pathway for the other components. Once the needle is positioned, the cannula is pushed downward over the needle until the entire opening is pushed out of the ventricle wall. Reaching this position, the surgeon can push out the apex pledget by means of the piston to form the apex fixation. When the apex pledget closes in the accordion shape, it creates a solid fixation for the implant, thus preventing bleeding after the extraction of the cannula.

#### Leaflet fixation unit

The apex fixation unit fits into a dedicated channel of a leaflet clamp, composed of two parts (6 and 7). The leaflet clamp is part of the leaflet fixation unit including also an external shell (4), a thread support (5), and a miniature harpoon-shaped needle (8). The artificial chord, at one side connected to the apex to the heart, is at the other side kept in position in the leaflet clamp by the thread support and the external shell. In order to fix the artificial chord to the leaflet, the ChoRe system is moved upward from the apex to the leaflet. Here, the leaflet is clamped between parts 6 and 7. The harpoon-shaped needle is then inserted and pushed through the leaflet to hook the artificial chord and to pull it up through the leaflet. The external shell is then rotated to release the artificial chord from the leaflet clamp.

#### Length adjustment unit

**Figure 4.**
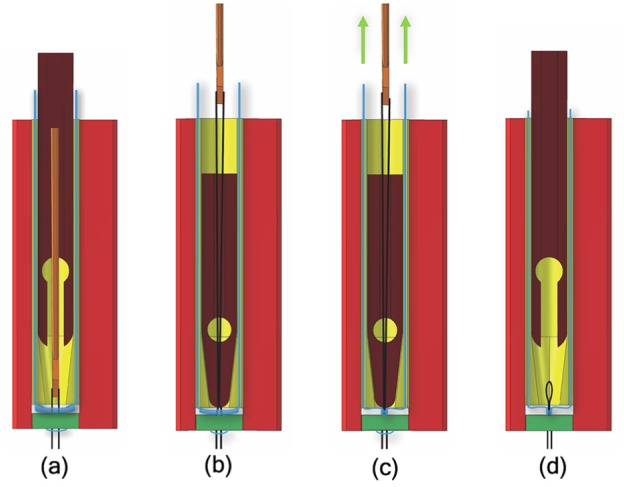
(a) The needle passes through the leaflet pledget after capturing the ePTFE thread; (b) the chord grasper closes around the ePTFE thread due to the clamping tube; (c) the pre-constructed knot is closed around the ePTFE thread to finally fix the implant; (d) the chord grasper releases the artificial chord, as well as the harpoon-shaped needle. In the sketches, the whole process is emphasized in displacements.

The length adjustment unit consists of a chord grasper (9), which can slide along the main axis into a clamping tube (10) (Figure 3(a)). The main role of these components is to enable the surgeon to tune the length of the artificial chord. When the artificial chord has been pulled up through the leaflet, the harpoon-shaped needle is positioned above the chord grasper and the clamping tube. The harpoon-shaped needle can be moved up and down relative to the leaflet to adjust the chord length under ultrasound imaging. During the adjusting, the chord grasper can be closed by pushing it out of the clamping tube, thereby temporarily fixing the chord and setting its length. At the correct chord length, the pre-constructed knot is fastened by pulling at both ends of the knot and the implant is completed. The two ends of the knot are then cut off once ChoRe is retracted (Figure 4).

**Figure 5.**
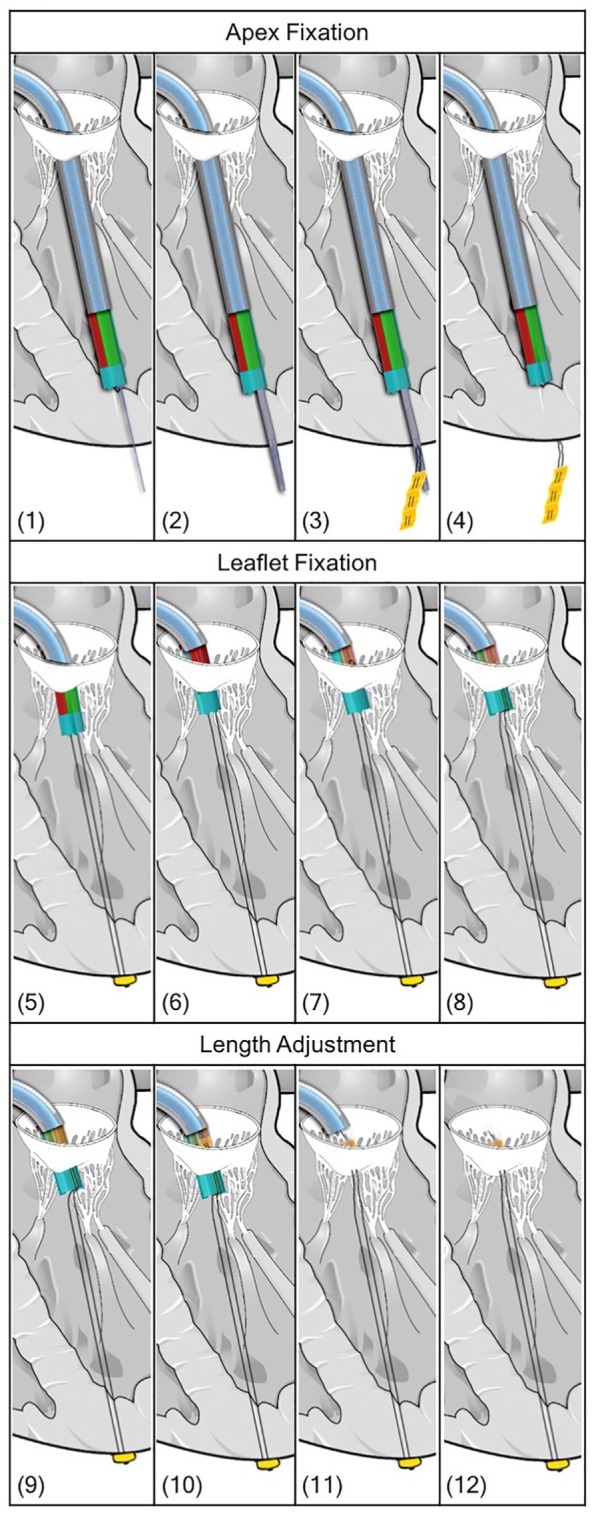
(1–12) Drawings of the procedure steps with the Solidworks model of the ChoRe device. The steerable catheter is represented in light blue.

The procedure described above can be summarized into the following 12 steps (Figure 5).

#### Apex fixation

1. The needle is pushed through the apex of the heart to create the pathway for the cannula.
2. The cannula slides over the needle until the opening is all out of the ventricle wall.
3. The apex pledget is pushed out through the opening by the piston.
4. The needle, the cannula, and the piston are retracted into the leaflet clamp.

#### Leaflet fixation

5. The ChoRe is moved up to reach the leaflet plane and the apex pledget folds to create the apex fixation.
6. The leaflet clamp grasps the leaflet.
7. The harpoon-shaped needle is pushed through the leaflet to hook the artificial chord and to pull it up through the leaflet, above the chord grasper and the clamping tube.
8. The external shell is rotated and the artificial chord is released from the leaflet clamp.

#### Length adjustment

9. The chord grasper can be closed around the artificial chord by pushing it out of the clamping, temporarily fix the length.
10. The pre-constructed knot is fastened by pulling at both ends of the knot.
11. The pre-constructed knot is left on the leaflet.
12. The harpoon-shaped needle releases the artificial chord due to a small movement toward the leaflet and a small rotation. The leaflet clamp is opened to release the leaflet and closed afterward to retract the entire device back in the steerable guiding sheath.

### Prototype manufacturing

**Figure 6.**
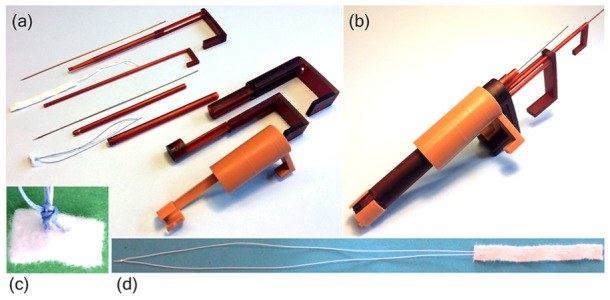
The 3-D-printed and scaled-up ChoRe prototype with the manipulator part: (a) The disassembled ChoRe prototype. The picture shows all the elements. Starting from the top: the needle, the cannula, the artificial chord, the piston, the harpoon-shaped needle, the chord grasper, the pre-constructed knot, the clamping tube, the leaflet clamp, and the external shell; (b) the assembled ChoRe prototype; (c) an example of the pre-constructed knot structure tightened around the artificial chord; (d) the artificial chord: the apex pledget and the ePTFE in loop configuration.

In order to evaluate its functionality, a ChoRe prototype has been constructed (Figure 6). Most parts were manufactured with additive manufacturing technology using the 3-D printer Perfactory®4 Mini XL with an Enhanced Resolution Module (ERM) provided by EnvisionTEC (Gladbeck, Germany) at TU Delft. The materials used to print the different parts are liquid photopolymers, R5 and R31, specifically customized for prototyping (Figure 6(a) and (b)). The RC31 material is stiffer than R5 and was used to make the external shell more rigid. In addition, the needle for the apex fixation and the harpoon-shaped needle were made of stainless-steel wire. All parts were manufactured twice as large as the designed size due to constraints related to the use of 3-D printer (0.06 × 0.044 mm native pixel size and 0.4 mm in resolution). All elements were printed as separate individual parts, except for the leaflet clamp in which the thread support was glued after the printing.

The geometrical design as described in these sections is the intended design for ChoRe to function in a trans-catheter scenario. Simple hand-pieces were added to operate the device during the test to investigate its functionality.

## Functional test in bovine hearts

### Parameters of interest

The functionality of the ChoRe prototype determines the success of the procedure and relies on the correct placing of the implant. The prototype was therefore tested ex-vivo in bovine hearts due to the scaled-up size of the device (twice as large as the final size). We qualitatively tested the outcome of the procedure by performing it 10 times. We also measured the time that is required to conduct the procedure to have a preliminary evaluation of the amount of time the final procedure would take.

### Material preparation

#### Artificial chords preparation

The test was carried out by using GoreTex ePTFE biocompatible suture thread (W. L. Gore &Associates, Inc., Arizona, USA)^44^ and Bard®PTFE felt fabric (Bard Peripheral Vascular, Inc., Arizona, USA) for the pledgets.^45^ ePTFE is a specific type of thread customized for the repair of the chordae tendineae; it is a highly flexible, non-absorbable, microporous monofilament with high resistance to fatigue and tensile strength, and high bio-integration property. Bard®PTFE felt is a non-absorbable fabric usually applied as buttresses under sutures to reinforce the tissue and avoid tearing, for instance, in the repair of left ventricle rupture.^46^ The pledget was cut in a rectangular element, folded into an accordion shape, and sewn together with the ePTFE thread, as shown in Figure 6(d), to allow its deployment. For the pre-constructed knot, a polyester thread (Medtronic, Minnesota, USA), and the Bard®PTFE felt were used. The polyester thread was sewn into the pledget and the pre-constructed knot was created as shown in Figure 6(c).

#### Bovine hearts preparation

The test was performed ex-vivo using bovine hearts. Bovine hearts are commonly used in cardiac studies due to their similarities with the human heart at a larger scale.^47^ The dissection of each heart was performed following the procedure used by Lodder et al.,^48^ thus to have a better visualization of the left ventricle. First, the right chambers of the heart were taken out, as well as the left atrium. Second, the inter-ventricular septum was removed. Then, a portion of the ventricle wall was removed to obtain a better visualization of the mitral valve structure. Finally, the natural chords were cut at the locations at which they commonly break. This included the chords connected to the free margin of the posterior leaflet.^49^

#### Test procedure

**Figure 7.**
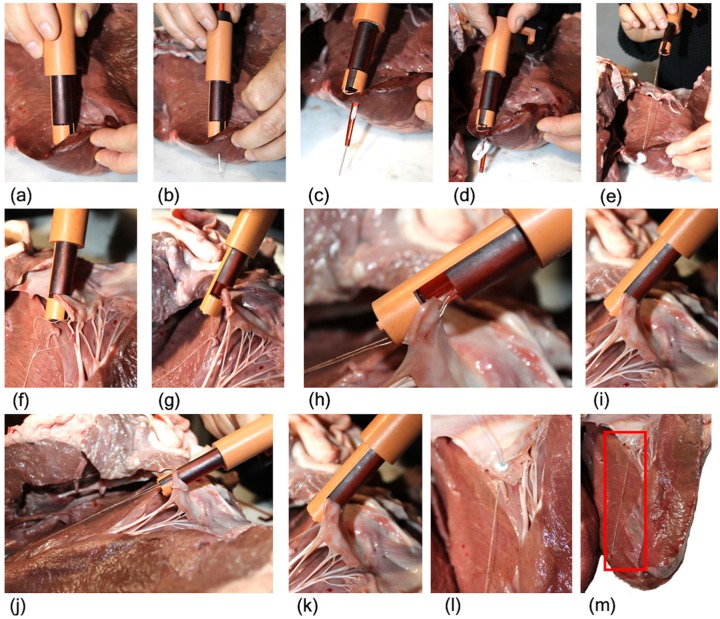
Procedural steps of the functional test carried out on bovine hearts: (a) ChoRe is positioned on the apex of the heart; (b) the needle is pushed through the apex; (c) the cannula is pushed through the apex; (d) the apex pledget is pushed out; (e) ChoRe is moved to the leaflet plane; (f) the leaflet is grasped; (g) the harpoon-shaped needle passes through the leaflet; (h) a detail with the clamp opened to show the harpoon-shaped needle after having captured the chord; (i) the chord length is fixed with the chord grasper; (j) the external shell is rotated; (k) the leaflet is released; (l) a detailed of the leaflet fixation of implanted chord; (m) the implanted chord, highlighted by the red square.

The experimental set-up comprised the bovine hearts and a chronometer. A total of 10 trials were performed to improve the statistic of the test. However, since the mitral valve structure of one heart has not enough space to accommodate 10 chords, we decided to use three bovine hearts, similar in size, and implant the artificial chords one after the other without removing the previously implanted chord. For practical reasons, a total of 10 cannulae were prepared in advance and loaded with pledgets for the apex side, while the pre-constructed knot was inserted into the leaflet clamp every time the procedure was repeated. The procedure was performed by one of the authors of this article with no experience with the ChoRe when started the functional test. The task completion time was measured with the chronometer. For each implant, the time measuring was started when the ChoRe was positioned in the starting position (in the apex of the left ventricle) and was stopped when the implant was finally attached to the leaflet. The overall procedure was performed according to the method described above (Figure 7).

## Results

**Figure 8.**
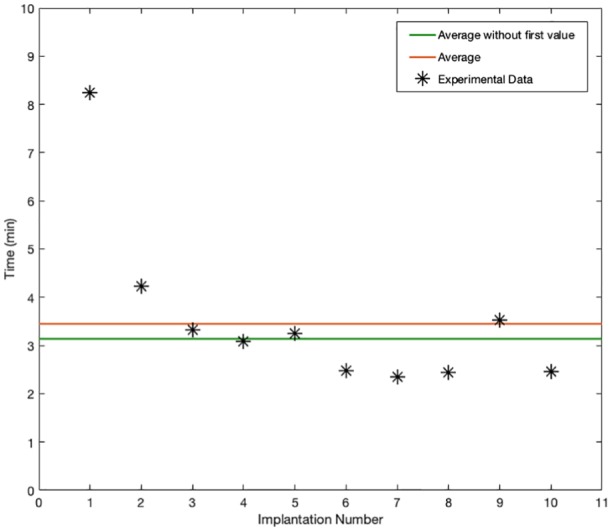
Plot of the duration of the procedure, in minutes, for each of the 10 trials (black stars). The red line shows the average of the duration considering all 10 trials while the green line shows the average without the first measurement.

During the test, all 10 artificial chords were successfully implanted. In all the trials, the ChoRe was able to attach the artificial chord first to the apex of the ventricle and then at the leaflet of the mitral valve. The maximum time registered during the first trial was 8.25 min, while the minimum time to perform the procedure was 2.34 min. The calculated average time was 3.45 ± 1.44 min considering all 10 trials and 3.14 ± 0.36 min without considering the first time (nine trials), as shown in Figure 8.

Figure 8 shows that a learning factor has a principal role in the collected data of the one chord fixation time. As the number of the performed procedures increased, the time decreased accordingly with an average of 3.45 ± 1.44 min considering all 10 trials (average of 10 values) and 3.14 ± 0.36 min without the first measurement (average of nine values). After six trials, the time value stabilizes with an average of 2.59 ± 0.35 min (average of the four last values) keeping constant the intra-variability of the data. These data give just an impression of the time the final procedure would take to implant one chord but can be compared to the average of 5 min for each implanted chord reported by Rucinskas et al.^26^ using the clinically accepted Neochord device in a trans-apical procedure.

## Discussion

### A new concept for chord replacement

This work presents a new device for the treatment of chordae tendineae rupture using trans-catheter approach. The compactness of ChoRe comes without any sharp edges. As such, the resulting device allows for easy motion into the cardiac chambers without creating damage. The simplicity of the design allows for relatively fast production time and assembly using 3-D printing technology for the functional test phase. Even though this first prototype of ChoRe has proven its functionality and overall performance, a number of improvements can be made to further develop the design. For example, the length of the opening in the cannula, in the first stage determined considering the thickness of the heart wall, may be designed longer in order to allow relatively easier ejection of the pledget. Another improvement involves the design of the piston, which could have a narrower tip to allow the pledget to be pushed out better. The need for these adjustments became apparent after the first prototype was manufactured and assembled.

The dimensions of ChoRe in its intended scale, 22 F in diameter and 25 mm in length, are relatively modest and similar to one of the devices currently used in mitral valve trans-catheter procedures.^40^ These dimensions leave the possibility to use the device in the majority of the patients, retaining its geometry. Moreover, to minimize risks of additional damages to the mitral valve structure, we tried to implement technologies already in use for heart surgery. For example, the harpoon-shaped needle was kept with the same size of the one already used in Neochord^50^ and the in accordion-shape pledget is already used in the heart to reduce the size of the annulus of the mitral valve in the Mitralgn system.^29^

To conclude, while commercially available devices, such as the Neochord,^50^ or devices still in a clinical trial phase, such as the TSD-5^22^ or the V-chordal off-Pump,^23^ are generally intended for trans-apical use, ChoRe was developed to be integrated into a catheter and perform the repair of the chordae tendineae in trans-catheter scenarios. Having the intent to use the device in trans-catheter procedures with a beating-heart condition in the future, the device needs to be further improved for catheterization possibilities; for example, the connection between the flexible shaft and the ChoRe needs to be developed, as well as the controls of each element of the device through a flexible catheter.

### Prototyping and testing

With the first prototype being created through additive manufacturing, the quality of the design could be evaluated quickly. This allows ameliorating aspects of the device during the manufacturing phase, such as the size of the chord grasper. Similarly, the test set-up, as well as the results, could be gained relatively fast. For example, it was noticed that the preloading of the cannula with the pledget took approximately 30 min on average. This time needs to be reduced to increase the performance of the device, for example, by means of a dedicated loading device. Even though manufacturing a 3-D-printed model is a relevant opportunity to analyze the device in multiple aspects, there are a number of disadvantages related to this method of manufacturing. The polymeric and resin materials that were used to print the device resulted in breakages during the test phase due to their brittle characteristics. In addition, it was not possible to print rigid needles due to their small diameters, which resulted in flexible components.

Analyzing the behavior of the prototype during our functional test, we found that the solution to connect the ePTFE thread at the apex of the heart seems to yield good results. This included the mechanical functionality of device components and the robustness of the apex pledget attachment. On the contrary, the upper side of the connection showed some weaknesses. The grasp of the leaflet with the leaflet clamp, the harpoon-shaped needle, and the length adjustment unit functioned according to the requirements, whereas the mechanism of fixation for the leaflet connection has to be re-designed as the pre-constructed knot. In fact, even though the mechanism of the fixation for the leaflet connection was able to fix the length of the artificial chord, it was not able to maintain the length and the point of anchorage when pulled. The reason for this can be found in the slippery properties of the ePTFE thread, as well as in the pre-constructed knot structure that required multiple loops of the thread around the artificial chord. A possible solution to eliminate these problems may be to replace the pre-constructed knot with a clip that is able to fix the thread to the leaflet side. Another important drawback concerns the fixation of a single artificial chord for each insertion of the device. Having a device that is able to place multiple chords during one device insertion could potentially reduce the time and complexity of the procedure.

Moreover, it has been shown that the length of the implanted ePTFE artificial chord affects the outcome of the repair. In the case of an apex connection, as reported by Grinberg et al.,^51^ the length of the artificial chord is approximately two times longer than the chords implanted in a conventional procedure, attached to the papillary muscles. Studies have shown that the increased length leads to a lack of potential shock-absorption and an increase in stiffness that can negatively affect the long-term results and lead to an early failure of the implant.^52,53^ Possible solutions, as suggested by Jensen et al.,^53^ could be to modify the trans-ventricular fixation point in order to reduce the length of the chords, or change the material of the artificial chord. ePTFE is a highly porous suture thread that facilitates the bio-integration of the implant; however, other types of suture threads could be an option due to the adaptability of our design that allows the use of different kinds of threads.

Future research will focus on improving not only ChoRe individual elements, such as the piston shape or the opening of the cannula, but also the functionality of the device, such as reducing the time for preloading the cannula. Moreover, the device will be adapted to be used in beating-heart condition and will be fabricated in its intended scale (22 F in diameter and 25 mm in length), with more resistant, ISO10993 biocompatible materials, such as AISI 316 or 304 stainless steel for the needles and polymers such as polyether ether ketone (PEEK) or polyethylenimine (PEI) for the printed parts, and integrated with a long, flexible catheter shaft that can be used during catheterization. Nevertheless, our ChoRe shows great potential in developing advanced surgical devices using 3-D printing technology and packing of complex multifunctionalities in a miniature device for mitral valve repair.

## Conclusion

This article introduced a novel mitral valve repair device that is intended to operate in a trans-catheter scenario. The device has been designed considering the constraints, given by the biological structures, and the fundamental procedure functions. The artificial chord must be attached to the lower side of the mitral valve structure on the apex of the heart and to the upper side on the prolapsed leaflet of the mitral valve. A research prototype was manufactured with dimensions that were twice the required size to conduct an early evaluation of the properties. The functionality of the prototype was qualitatively tested giving a preliminary evaluation of the procedure time. The resulting data showed that, even if improvements in the design must be done especially for the connection of the artificial chord at the leaflet side in order to guarantee a stable connection, this first prototype of ChoRe is a good starting point for the development of new, multi-functional technology for integrated mitral valve repair. Future work will focus on the integration of the mitral valve repair device with a steerable catheter and a dedicated external manipulating device. This allows a future potential for more patient-specific and less-invasive treatments in mitral valve repair.
